# Valproic Acid Inhibits Ferroptosis and Improves Bone Integration in OVX Rats Through the AMPK/SIRT1 Pathway

**DOI:** 10.1002/kjm2.70175

**Published:** 2026-02-04

**Authors:** Qing‐Song Gu, Yi‐Fan Gu, Jian‐Qiao Li, Yi‐Heng Li, Yu‐Hu Chen, Lin‐Hui Wang, Zi‐Ru Wang, Yi‐Cong Wang, Min Yang

**Affiliations:** ^1^ Department of Trauma Orthopedics The First Affiliated Hospital of Wannan Medical College, Yijishan Hospital Wuhu Anhui People's Republic of China

**Keywords:** bone integration, bone marrow mesenchymal stem cells, ferroptosis, osteoporosis, valproic acid

## Abstract

The prevalence of postmenopausal osteoporosis (PMOP) has been steadily increasing. Ferroptosis has been recognized as a critical factor influencing the bone‐forming ability of bone marrow mesenchymal stem cells (BMSCs). Valproic acid (VPA), an HDAC inhibitor, has been suggested to play a role in regulating osteoporosis development; however, its underlying mechanism remains unclear. This study aims to explore the impact of VPA on ferroptosis, a process that is triggered by Erastin, and to assess its implications for postmenopausal osteoporosis (PMOP). We evaluated the effects of valproate sodium on Erastin‐induced ferroptosis in BMSCs through in vitro experiments, including CCK‐8 assays, Western blot analysis, mitochondrial function assessments (MDA, GSH, ROS, and MMP), and osteogenic evaluations (ALP and ARS staining). The impact of VPA on bone integration in ovariectomized (OVX) rats was assessed using micro‐CT, hematoxylin–eosin (HE) staining, Masson's trichrome staining, and RT‐PCR analysis. RNA sequencing was employed to investigate the underlying mechanisms of VPA action. Our findings demonstrate that VPA treatment prevents the Erastin‐induced decline in the osteogenic capacity of BMSCs. In addition, VPA treatment suppresses ferroptosis, as indicated by decreased malondialdehyde levels, reduced mitochondrial ROS, and increased glutathione concentrations. Moreover, VPA treatment promotes trabecular bone growth and enhances bone integration. Mechanistic studies reveal that SIRT1–siRNA counteracts the beneficial effects of VPA in Erastin‐treated BMSCs. VPA improves bone integration in OVX rats by activating the AMPK/SIRT1 pathway and inhibiting ferroptosis.

## 1 Introduction

With the ongoing global demographic aging, the prevalence of PMOP has been escalating, posing a growing, and severe public health challenge [1]. Osteoporosis not only significantly reduces the quality of life for patients but is also often accompanied by fractures, widespread pain, and psychological disorders [2, 3]. In patients with osteoporotic fractures, the integration of metal implants (such as titanium alloys) with bone tissue is often poor, increasing the risk of aseptic loosening of the implants [4]. Currently, osteoporosis treatments primarily aim to inhibit bone loss or promote increased bone density [5]. However, these treatments have several limitations, including limited efficacy, high costs, and potential significant side effects [6]. Therefore, gaining deeper insights into the molecular pathways underlying osteoporosis and designing more effective therapeutic solutions is essential.

Unlike apoptosis and necrosis, ferroptosis is a distinct form of cell death caused by iron metabolism disorders [7]. During ferroptosis, there is an accumulation of iron ions, along with lipid peroxidation and an increase in reactive oxygen species (ROS) levels [8]. The cystine/glutamate transporter (SLC7A11) and glutathione peroxidase 4 (GPX4) play crucial roles in inhibiting lipid peroxidation in cells [9]. The ferroptosis inhibitor Ferrostatin‐1 (Fer‐1) effectively prevents the progression of ferroptosis by regulating intracellular iron metabolism and oxidative stress [10]. Research has shown that ferroptosis is closely linked to the progression of various diseases, including tumors, neurological disorders, cardiovascular diseases, autoimmune diseases, and osteoporosis [11, 12, 13]. Additionally, ferroptosis may influence the progression of many estrogen‐related diseases [14]. Therefore, ferroptosis may play a critical role in the pathogenesis of PMOP, and targeting ferroptosis could become an effective therapeutic approach for treating estrogen deficiency‐related diseases, including PMOP.

Valproic acid (VPA) is a widely prescribed drug for managing epilepsy, seizures, and mood disorders [15, 16]. Recent studies have revealed that long‐term use of VPA may exert dual effects on skeletal health. On one hand, VPA is strongly associated with reduced bone mineral density (BMD) and increased risk of osteoporotic fractures [17]. On the other hand, VPA exhibits potential osteogenic effects under specific conditions. For instance, in a rat model of glucocorticoid‐induced femoral head necrosis, VPA significantly increased bone formation and reduced bone loss by enhancing osteoblast proliferation and differentiation [18]. Furthermore, VPA promotes osteogenic differentiation of murine bone marrow mesenchymal stem cells (BMSCs) and upregulates the expression of osteogenic markers such as osteocalcin [19]. These properties provide a theoretical foundation for VPA application in osteoporosis management. Emerging evidence highlights novel mechanisms of VPA in regulating cell death. Studies have confirmed VPA's ability to inhibit ferroptosis in renal tubular epithelial cells [20], yet its potential to ameliorate PMOP through ferroptosis regulation remains unexplored. To address this knowledge gap, the present study established BMSC ferroptosis models and OVX rat bone integration models, aiming to investigate VPA's regulatory effects on BMSC ferroptosis and bone integration, thereby providing new insights for targeted osteoporosis therapies.

This study was designed to investigate whether VPA ameliorates implant osseointegration in ovariectomized (OVX) rats, to determine the involvement of ferroptosis in this process, and to elucidate the role of the AMPK/SIRT1 signaling axis in mediating the observed effects.

## 2 Materials and Methods

### 2.1 Reagents and Instruments

The chemical reagents used in this study included dimethyl sulfoxide (DMSO, Sigma‐Aldrich, USA), VPA, Erastin, and Fer‐1 (MCE, USA). The media and supplements included DMEM‐F12 medium and fetal bovine serum (FBS, Thermo Fisher Scientific, USA), phosphate‐buffered saline (PBS, Macklin, Shanghai), and 0.25% trypsin–EDTA and penicillin–streptomycin solution (Procell, China). The primary antibodies used were GPX4, SLC7A11, BMP2, OPN, RUNX2, AMPK, SIRT1, and β‐actin (Abcam, UK). The secondary antibody was goat anti‐rabbit IgG (Thermo Fisher Scientific, USA). Other reagents included ascorbic acid, β‐glycerophosphate, and dexamethasone (Macklin, China). The assay kits used in the study included alkaline phosphatase (ALP) activity assay kit, alizarin red S (ARS) solution, CCK‐8 cell counting kit, and ROS assay kit (Beyotime, China); malondialdehyde (MDA) assay kit and glutathione (GSH) activity assay kit (Solarbio, China); BCA protein assay kit (Thermo Fisher Scientific, USA); hematoxylin and eosin (H&E) staining kit (Boster, China); and Masson's trichrome staining kit (Beyotime, China). The experimental equipment included an inverted fluorescence microscope (Nikon, Japan), a microplate reader (Bio Tek Instruments, USA), and a flow cytometer (Beckman Coulter, USA).

### 2.2 Isolation, Culture, and Osteogenic Differentiation of BMSCs

First, SD rats were sacrificed, and the femurs were aseptically excised. The bones were rinsed with PBS, and the epiphyses were detached. The bone marrow cavity was accessed using a sterile syringe and repeatedly flushed with culture medium to collect the cell suspension, which was subsequently centrifuged to isolate the cells. The extracted cells were resuspended and transferred into culture flasks. After 48 h, the culture medium was refreshed, and the cells were subcultured when they reached the appropriate density. The experiment was conducted using bone marrow‐derived mesenchymal stem cells (BMSCs) at passages 3–5. The culture medium used for cell maintenance was DMEM/F12, containing 10% FBS and 1% penicillin–streptomycin. Cells were cultured at 37°C in a humidified atmosphere with 5% CO_2_. The medium was refreshed every 2 days, and subculturing was performed once cell confluence reached approximately 80%. For osteogenic differentiation, the cells were exposed to DMEM/F12 supplemented with 10% FBS, 1% penicillin–streptomycin, 10 mM β‐glycerophosphate, 50 μM ascorbic acid, and 100 nM dexamethasone.

### 2.3 Cell Viability Assay

Cell viability was assessed using a CCK‐8 assay kit, following the manufacturer's instructions. After drug treatment, 10 μL of CCK‐8 reagent was added to each well and incubated for 1 h. The absorbance (OD) at 450 nm was then recorded using a microplate reader.

### 2.4 Alkaline Phosphatase (ALP) Staining and Activity Assay

Cells were cultured in a cyclic manner, with 2 days in osteogenic differentiation medium followed by 1 day of group‐specific treatment, repeated every 3 days. After 7 days of culture, cells were fixed with 4% paraformaldehyde for 10 min at room temperature. ALP staining was performed using BCIP/NBT solution (Solarbio, China) under light‐protected conditions for 40 min, followed by microscopic observation and image acquisition. For quantitative ALP activity measurement, cells were lysed, and the enzymatic activity was determined using a commercial ALP assay kit (Sigma‐Aldrich, USA) according to the manufacturer's protocol. Absorbance was measured at 405 nm using a microplate reader (BioTek, USA), with results normalized to total protein concentration determined by a BCA assay.

### 2.5 Alizarin Red S (ARS) Staining and Mineralization Quantification

Following 21 days of osteogenic induction, BMSCs were washed with PBS and fixed with 4% paraformaldehyde for 20 min at room temperature. Cells were then stained with 2% Alizarin Red S solution (pH 4.2, Sigma‐Aldrich) at 37°C for 30 min. After PBS washing, calcium nodule formation was visualized under an inverted microscope (Nikon Eclipse Ti, Japan). For quantitative analysis, stained cells were incubated with 10% cetylpyridinium chloride (CPC, Sigma‐Aldrich) for 60 min at room temperature, and absorbance was measured at 570 nm using a microplate reader (BioTek Synergy H1, USA).

### 2.6 GSH Activity Assay

GSH levels were determined using a commercial kit (A006‐2, Nanjing Jiancheng Bioengineering Institute, China) following the manufacturer's protocol. Briefly, cells were washed three times with PBS and lysed on ice. After centrifugation at 12,000*g* for 10 min at 4°C, 50 μL supernatant was mixed with chromogenic reagent and incubated in the dark for 20 min. Absorbance was measured at 412 nm and normalized to total protein concentration.

### 2.7 MDA Content Measurement

MDA levels were assessed using a lipid peroxidation assay kit (A003‐1, Nanjing Jiancheng Bioengineering Institute). Samples were mixed with thiobarbituric acid (TBA) reagent, heated at 95°C for 30 min, and centrifuged at 4000*g* for 10 min. The supernatant absorbance was measured at 530 nm and normalized to protein content.

### 2.8 ROS Detection

Intracellular ROS levels were measured using the DCFH‐DA probe (KGAF019, KeyGEN BioTECH, China). Cells were incubated with 10 μM DCFH‐DA at 37°C for 40 min in the dark, washed with PBS, and analyzed by fluorescence microscopy (Olympus IX83, Japan) and flow cytometry (BD FACSCanto II, USA). Fluorescence intensity (Ex/Em: 488/525 nm) was quantified using FlowJo software (v10.6.2).

### 2.9 Mitochondrial Membrane Potential (MMP) Assessment

MMP was evaluated using the JC‐1 probe (C2006, Beyotime, China). Cells were stained with 5 μg/mL JC‐1 for 30 min at 37°C, followed by DAPI nuclear counterstaining. Fluorescence images were captured using a confocal microscope (Zeiss LSM 880, Germany). The red/green fluorescence ratio was calculated using ImageJ software (NIH, USA).

### 2.10 Transmission Electron Microscopy (TEM) Analysis

Cells were fixed with 2.5% glutaraldehyde in 0.1 M PBS (pH 7.4) at 4°C for 24 h, post‐fixed with 1% osmium tetroxide for 1 h, and dehydrated through an ethanol gradient. Samples were embedded in Epon 812 resin, sectioned (70 nm), and stained with uranyl acetate and lead citrate. Ultrastructure was examined using a TEM (Hitachi HT7800, Japan) at 80 kV.

### 2.11 siRNA Transfection

SIRT1 siRNA (siB1505161036, GeneChem, China) was transfected into BMSCs at 50%–60% confluency using Lipofectamine 3000 (L3000015, Invitrogen, USA). After 4–6 h incubation, the medium was replaced with fresh complete medium. Transfection efficiency (> 70% knockdown) was confirmed by Western blot after 48 h. Cell viability was > 98% at 12 h post‐transfection (CCK‐8 assay).

### 2.12 Experimental Design and Surgical Procedures

Ethical approval was obtained from the Ethics Committee of Yijishan Hospital. Fifty female SPF Sprague–Dawley rats (8 weeks old, 180 ± 20 g) were housed in the Central Laboratory of Yijishan Hospital. Twenty animals were reserved for BMSC isolation, and the remaining 30 were used for in vivo experiments. After a 1‐week acclimatization period under standard conditions (12 h light/dark cycle, 20°C–24°C, 50% humidity, ad libitum food and water), osteoporosis was induced by bilateral ovariectomy (OVX). Sham‐operated animals underwent resection of the periovarian fat only. All rats were allowed unrestricted cage activity for 3 months to establish the osteoporotic phenotype.

Model validation was performed thereafter:

BMSCs were harvested from the femora of 20 rats (*n* = 10 Sham, *n* = 10 OVX) for subsequent in vitro experiments.

The remaining 30 rats received a standardized unicortical defect (2 mm) in the left distal femur followed by titanium‐pin implantation and were randomly allocated to three groups (*n* = 10 each):

Con group: Sham + defect + vehicle (normal saline, gavage every other day).

OVX group: OVX + defect + vehicle (gavage every other day).

VPA group: OVX + defect + VPA (150 mg kg^−1^, gavage every other day).

Eight weeks after implantation, all animals were euthanized. The left femora were subjected to micro‐CT analysis; the right femora were collected for histopathology, Western blot, and RNA‐sequencing analyses.

### 2.13 Micro‐CT Analysis

The titanium rod‐implanted femur region was scanned using a SkyScan 1176 system (Bruker, Belgium) at 70 kV, 200 μA, 300 ms exposure time, and 15 μm resolution. Three‐dimensional (3D) reconstruction and analysis of bone parameters (BV/TV, Tb.N, Tb.Th, Tb.Sp, and BMD) were performed using CTAn software (v1.18.8.0). A 2 mm × 2 mm cylindrical volume of interest, centered on the titanium screw and located 1–3 mm distal to the growth plate in the femoral metaphysis, was scanned. A total of 300 continuous slices (10 μm thickness) were acquired; the middle 200 slices were reconstructed for 3D analysis by an operator blinded to group allocation.

### 2.14 Histological Staining (H&E and Masson's Trichrome)

Decalcified femurs were paraffin‐embedded and sectioned at 5 μm thickness. Sections were stained with H&E or Masson's trichrome following standard protocols. Bone trabeculae (H&E: pink/red; Masson: blue) were imaged using a digital slide scanner (Pannoramic 250, 3DHISTECH, Hungary). A standardized cancellous region in the distal metaphysis (1–3 mm below the growth plate, 2 mm × 2 mm field centered on the screw) was analyzed. Three sections per animal were examined; four random fields per section (×10 objective) were captured, yielding 12 images for trabecular morphometry.

### 2.15 Immunohistochemistry (GPX4)

Sections were deparaffinized, rehydrated, and subjected to antigen retrieval in citrate buffer (pH 6.0). After blocking with 5% BSA, sections were incubated with anti‐GPX4 antibody (ab125066, Abcam, 1:200) overnight at 4°C, followed by HRP‐conjugated secondary antibody and DAB development. Counterstaining was performed with hematoxylin. Quantification was performed using ImageJ (NIH, USA) on five random fields per sample.

### 2.16 Western Blot Analysis

Protein samples were prepared by lysing cells in RIPA buffer (P0013B, Beyotime) containing protease inhibitors. Protein concentrations were determined using a BCA assay kit (23,225, Thermo Fisher Scientific). Samples were mixed with 4× Laemmli buffer (1610747, Bio‐Rad) at a 4:1 ratio and denatured at 100°C for 10 min. Proteins (20–50 μg per lane) were separated on 10% SDS‐PAGE gels at 80 V for 30 min followed by 120 V for 70 min in running buffer (25 mM Tris, 192 mM glycine, 0.1% SDS). Proteins were transferred to PVDF membranes (IPVH00010, Millipore) activated in methanol, using a wet transfer system at 120 V for 60 min in ice‐cold transfer buffer (25 mM Tris, 192 mM glycine, 20% methanol). Membranes were blocked with 5% non‐fat milk in TBST (20 mM Tris–HCl, 150 mM NaCl, 0.1% Tween‐20) for 2 h at room temperature. After incubation with primary antibodies (1:1000 dilution in 5% BSA/TBST) overnight at 4°C and HRP‐conjugated secondary antibodies (1:5000) for 2 h at room temperature, protein bands were visualized using ECL reagent (32106, Thermo Fisher Scientific) and quantified using ImageJ software (NIH). All primary antibodies used for Western blot were purchased from Affinity Biosciences (Cincinnati, OH, USA) and were applied at the following dilutions and catalogue numbers: β‐actin (AF7018, 1:10,000), BMP2 (AF5163, 1:1000), OPN (AF0227, 1:1000), Runx2 (AF5186, 1:1000), GPX4 (DF6701, 1:800), SLC7A11 (DF12571, 1:800), total AMPKα (AF6423, 1:1000), phospho‐AMPKα Thr172 (AF3423, 1:1000), and SIRT1 (DF6033, 1:1000). The corresponding HRP‐conjugated goat anti‐rabbit or anti‐mouse IgG secondary antibodies (catalogue numbers S0001/S0002, Affinity Biosciences) were used at 1:5000 dilution.

### 2.17 Quantitative Real‐Time PCR (qRT‐PCR)

Total RNA was extracted using TRIzol reagent (15596026, Thermo Fisher Scientific) and reverse transcribed into cDNA using a PrimeScript RT reagent kit (RR047A, Takara). qPCR was performed using SYBR Premix Ex Taq (RR420A, Takara) on a QuantStudio 6 system (Thermo Fisher Scientific) with the following primers: GAPDH: F: 5′‐TGCACCACCAACTGCTTAG‐3′, R: 5′‐GGCATGGACTGTGGTCATG‐3′ RUNX2: F: 5′‐CAGGTGGAAGAGTGGAGGA‐3′, R: 5′‐AGCCAGGACCAATGACATG‐3′ OPN: F: 5′‐GAGGGAGTGTGTGGAAGAA‐3′, R: 5′‐GCCAGTACCTGTCCTTCTG‐3′ Relative gene expression was calculated using the 2^−ΔΔ*C*t^ method with GAPDH as the internal control.

### 2.18 Ferrous Iron (Fe^2+^) Content Assay

Tissue lysates were mixed with potassium permanganate solution and incubated at 60°C for 1 h. After adding chromogenic reagent and incubating at room temperature for 20 min, samples were centrifuged at 12,000*g* for 10 min. Supernatant absorbance was measured at 520 nm using a microplate reader (BioTek). Fe^2+^ concentration was calculated using a standard curve.

### 2.19 MDA Assay

Tissue homogenates were mixed with TBA reagent and heated at 95°C for 30 min. After centrifugation at 4000*g* for 10 min, supernatant absorbance was measured at 530 nm. MDA content was normalized to protein concentration determined by BCA assay and expressed as μmol/mg protein.

### 2.20 RNA Sequencing

Total RNA was extracted using TRIzol reagent, and mRNA was enriched using poly‐T magnetic beads. Sequencing libraries were prepared following the manufacturer's protocol and quality control was performed by Personal Biotechnology Co. Ltd. (Shanghai). Differentially expressed genes were identified with thresholds of greater than or equal to twofold change and adjusted *p* < 0.05.

### 2.21 Statistical Analysis

Data are presented as mean ± SD. Statistical analyses were performed using GraphPad Prism 8.0 (GraphPad Software). Comparisons between two groups were analyzed by unpaired Student's *t*‐test, while multiple group comparisons were evaluated by one‐way ANOVA with Tukey's post hoc test. A *p* < 0.05 was considered statistically significant.

## 3 Results

### 3.1 Isolation and Identification of Rat BMSCs

**FIGURE 1:**
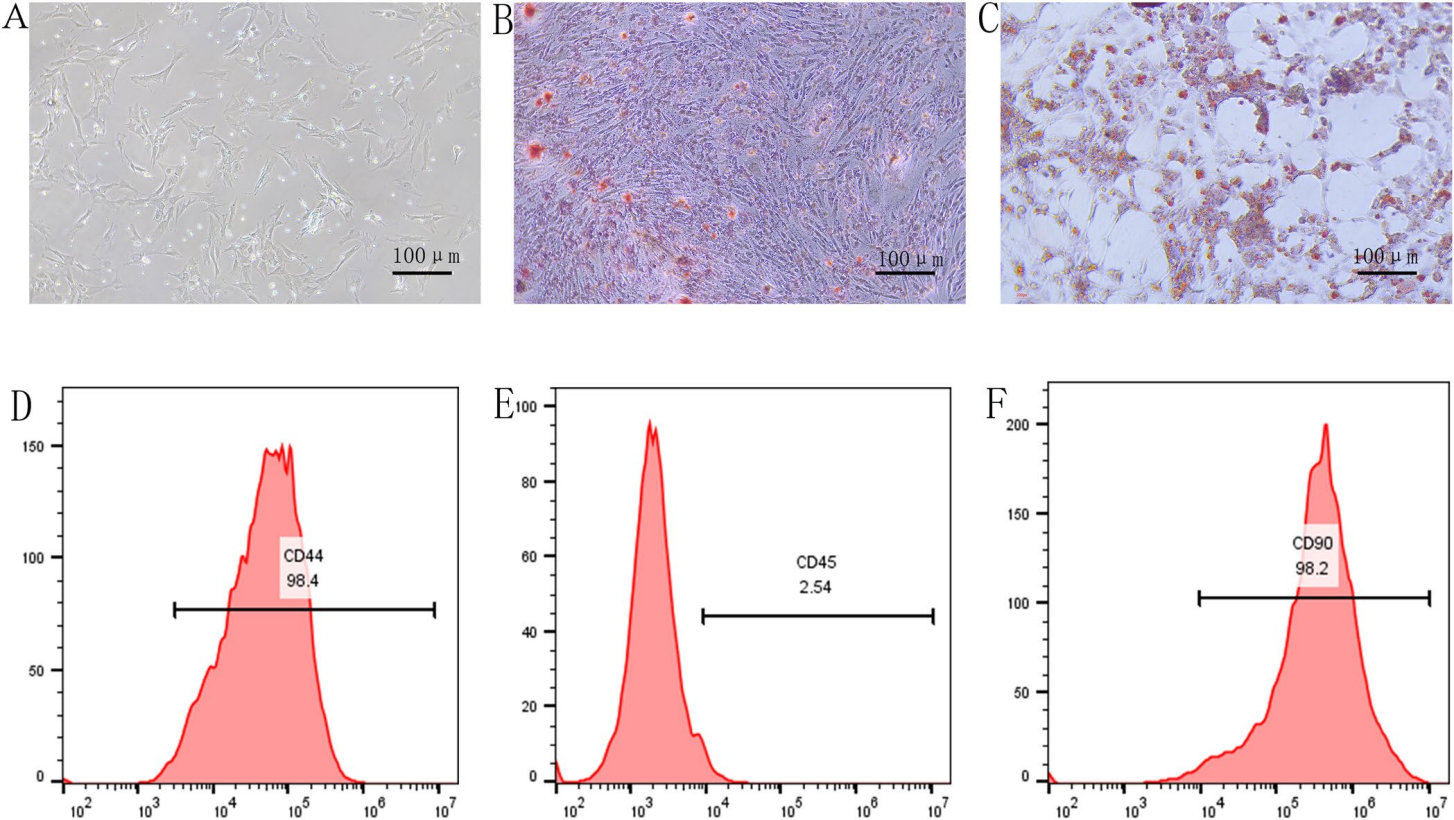
Differentiation and characterization of BMSCs. (A) BMSCs exhibited a spindle‐shaped morphology during in vitro culture. (B) Alizarin Red staining following osteogenic stimulation revealed calcium salt deposition, with a scale bar of 100 μm. (C) Oil Red O staining following adipogenic induction confirmed the formation of lipid droplets (scale bar = 100 μm). (D) Flow cytometry analysis revealed positive expression of surface markers CD44 and CD90, while CD45 expression was absent in BMSCs. All data presented were obtained from three independent experimental replicates.

Under in vitro culture conditions, the BMSCs were observed to exhibit a spindle‐shaped morphology (Figure 1A). To assess their differentiation potential, we induced their differentiation into osteoblasts and adipocytes. Alizarin red staining (Figure 1B) revealed orange‐red calcium deposits, while Oil Red O staining (Figure 1C) demonstrated orange‐red lipid droplets. Flow cytometric analysis showed high expression of CD44 and CD90 on the cell surface (positive rate > 85%), while CD45 expression was low (positive rate < 3%) (Figure 1D). These results confirmed that the isolated cells were BMSCs.

### 3.2 VPA Alleviates Erastin‐Induced Ferroptosis in BMSCs

**FIGURE 2:**
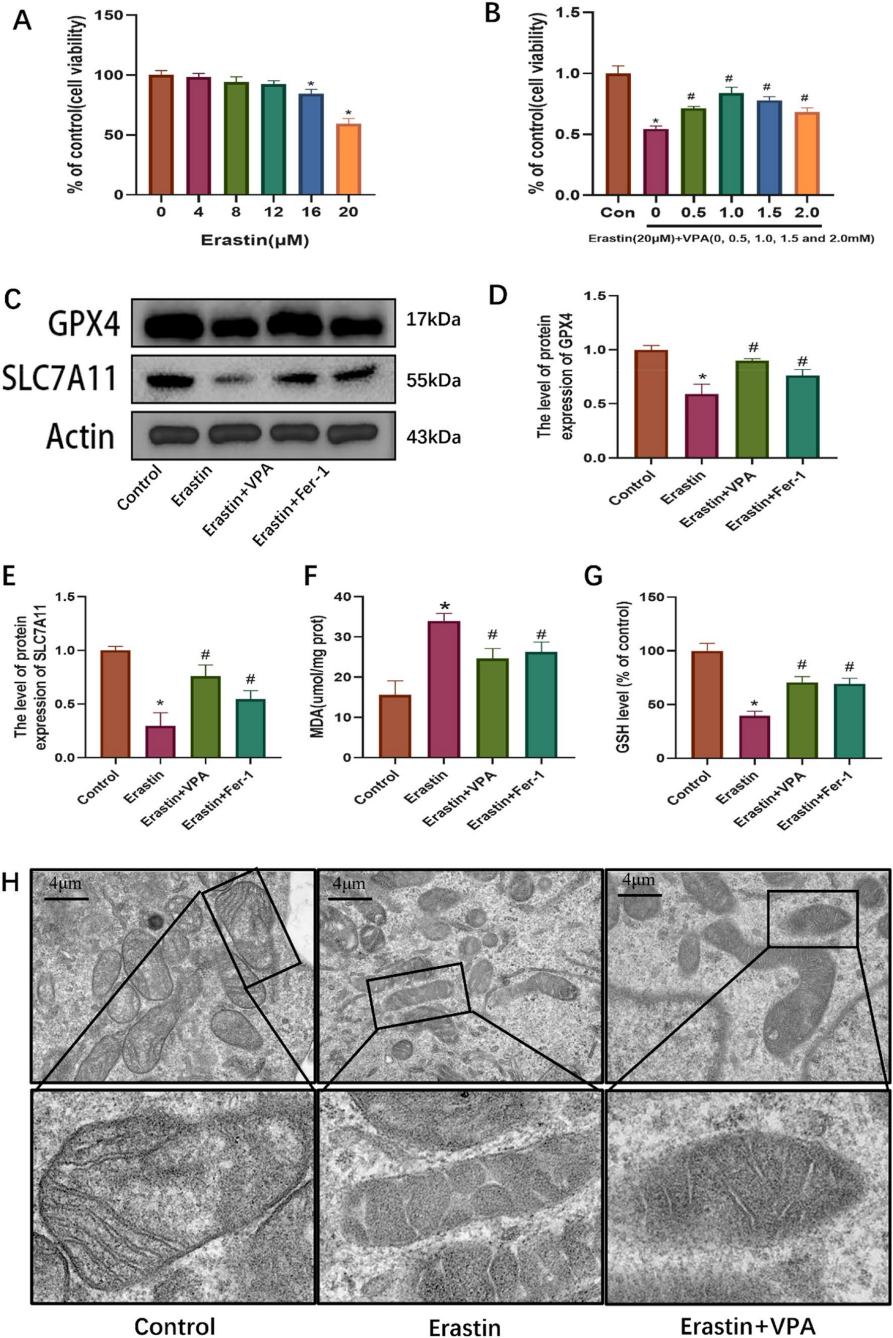
VPA attenuates Erastin‐induced ferroptosis in BMSCs. (A) Viability of BMSCs treated with different concentrations of Erastin (4, 8, 12, 16, and 20 μM) for 24 h. (B) Effect of VPA at varying concentrations (0.5, 1.0, 1.5, and 2.0 mM) on the viability of BMSCs treated with 20 μM Erastin. (C–E) Representative Western blot bands and relative expression levels of ferroptosis‐related proteins (GPX4 and SLC7A11). (F) Quantitative analysis of malondialdehyde (MDA) content. (G) Quantitative assessment of glutathione (GSH) levels. (H) Representative transmission electron microscopy (TEM) images. Data are presented as mean ± SD from three independent experiments. *p < 0.05 versus control group; ^#^ *p* < 0.05 versus Erastin‐treated group.

Under treatment with different concentrations of Erastin (4, 8, 12, 16, and 20 μM), cell viability showed a clear dose‐dependent decrease (Figure 2A). In our study, we observed that the combination of 20 μM Erastin with varying concentrations of VPA (0.5, 1.0, 1.5, and 2.0 mM) resulted in enhanced cell survival, with the most significant effect noted at a concentration of 1 mM (Figure 2B), corroborating findings from previous research that have shown the protective effects of VPA against Erastin‐induced cell damage. After determining the optimal concentration of VPA, we further investigated its inhibitory effect on Erastin‐induced ferroptosis. Western blot analysis revealed that GPX4 and SLC7A11 expression levels were markedly reduced in the Erastin‐treated group. However, these markers were restored following treatment with either VPA or Fer‐1 (10 μM) (Figure 2C–E). MDA and GSH levels, indicators of lipid ROS production, were notably increased and decreased, respectively, after Erastin treatment. Nonetheless, both VPA and Fer‐1 effectively mitigated lipid peroxidation (Figure 2F,G). The principal morphological characteristics of ferroptosis include mitochondrial shrinkage, increased membrane density, and reduced cristae. TEM analysis revealed that BMSCs treated with Erastin exhibited typical ferroptotic alterations, including shrunken mitochondria with ruptured outer membranes and diminished cristae. Notably, VPA treatment partially ameliorated these ultrastructural abnormalities, demonstrating moderate preservation of mitochondrial architecture (Figure 2H). Collectively, these results suggest that VPA effectively inhibits Erastin‐induced ferroptosis in BMSCs.

### 3.3 VPA Mitigates Erastin‐Induced Mitochondrial Dysfunction in BMSCs

**FIGURE 3:**
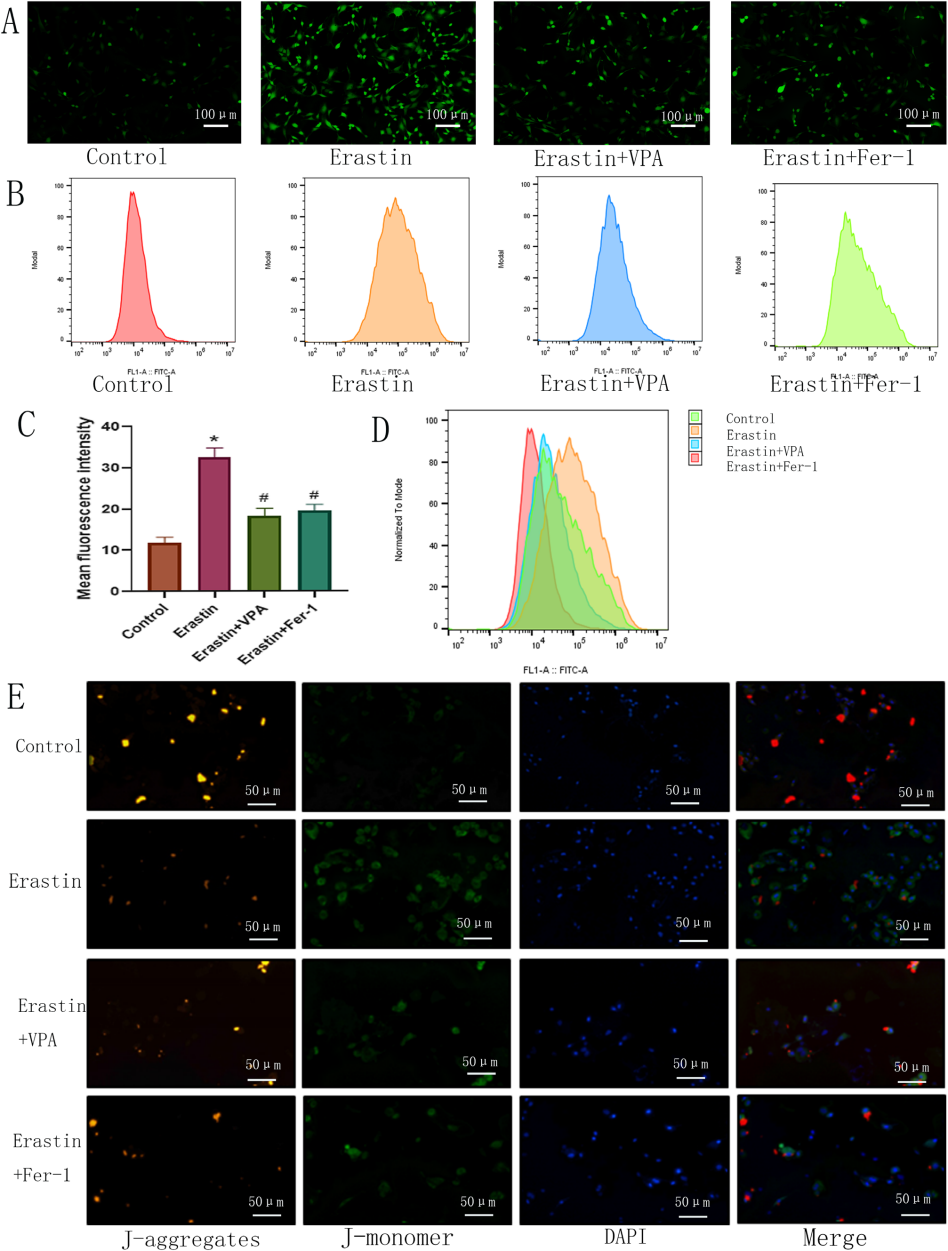
VPA ameliorates Erastin‐induced mitochondrial dysfunction in BMSCs. (A) Representative fluorescence microscopy images of DCFH‐DA‐labeled ROS (scale bar = 100 μm). (B) Flow cytometric analysis of intracellular ROS levels. (C) Quantitative analysis of mean fluorescence intensity (MFI) of ROS using ImageJ software. (D) Statistical comparison of fluorescence intensity among groups analyzed by FlowJo software. (E) JC‐1 probe assay demonstrating the effects of different treatments on mitochondrial membrane potential (MMP) in BMSCs. Data are presented as mean ± SD from three independent biological replicates. *p < 0.05 versus control group; ^#^ *p* < 0.05 versus Erastin‐treated group.

During ferroptosis, Erastin‐induced mitochondrial oxidative stress frequently results in mitochondrial dysfunction. The levels of ROS were detected using the DCFH‐DA probe, and the findings revealed a significant elevation in ROS levels following Erastin treatment, consistent with previous studies that have shown Erastin's role in inducing ferroptosis and increasing ROS production. Following treatment with either VPA or Fer‐1, a significant reduction in ROS levels was observed, as indicated by Figure 3A,C. Flow cytometry further validated this trend (Figure 3B,D). Additionally, MMP was evaluated using the JC‐1 probe. After stimulation with Erastin, a reduction in red fluorescence and an increase in green fluorescence indicated a loss of membrane potential. Nevertheless, treatment with VPA or Fer‐1 effectively restored both fluorescence intensities (Figure 3E). These results suggest that VPA successfully mitigates mitochondrial dysfunction.

### 3.4 VPA Enhances Osteogenic Potential of BMSCs by Modulating Ferroptosis

**FIGURE 4:**
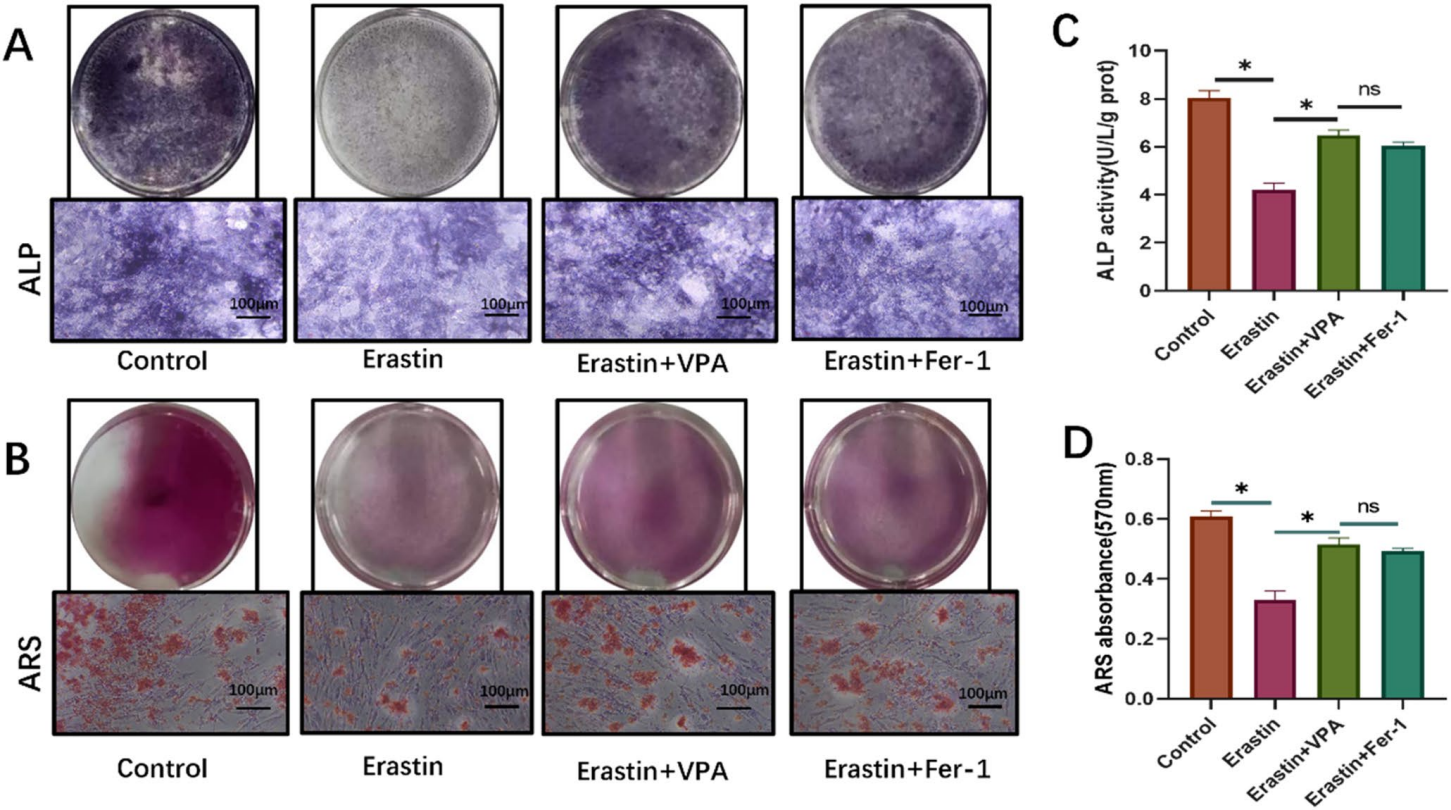
VPA enhances osteogenic potential of BMSCs by inhibiting ferroptosis. (A) Alkaline phosphatase (ALP) staining images of four experimental groups at Day 7 (scale bar = 100 μm). (B) Alizarin Red S (ARS) staining images showing mineralization nodules at Day 21 (scale bar = 100 μm). (C) Quantitative analysis of ALP activity among the four groups. (D) Spectrophotometric quantification of extracellular mineralization nodules at 570 nm. Data represent mean ± SD from three independent biological replicates. *p < 0.05; ns, not significant.

To explore the impact of ferroptosis on the osteogenic differentiation of BMSCs and assess the role of VPA in cells exposed to Erastin, we conducted the following experiments. Following 7 days of osteogenic induction, the administration of Erastin resulted in a significant decrease in ALP activity, as evidenced by Figure 4A,C, and inhibited the formation of calcium nodules by the 21st day, as shown in Figure 4B,D. Importantly, treatment with either VPA or Fer‐1 (10 μM) [21] significantly restored ALP activity and enhanced the mineralization capacity of BMSCs exposed to Erastin.

### 3.5 Molecular Docking Simulations Reveal Strong Binding Interactions Between VPA and the AMPK/SIRT1 Pathway

**FIGURE 5:**
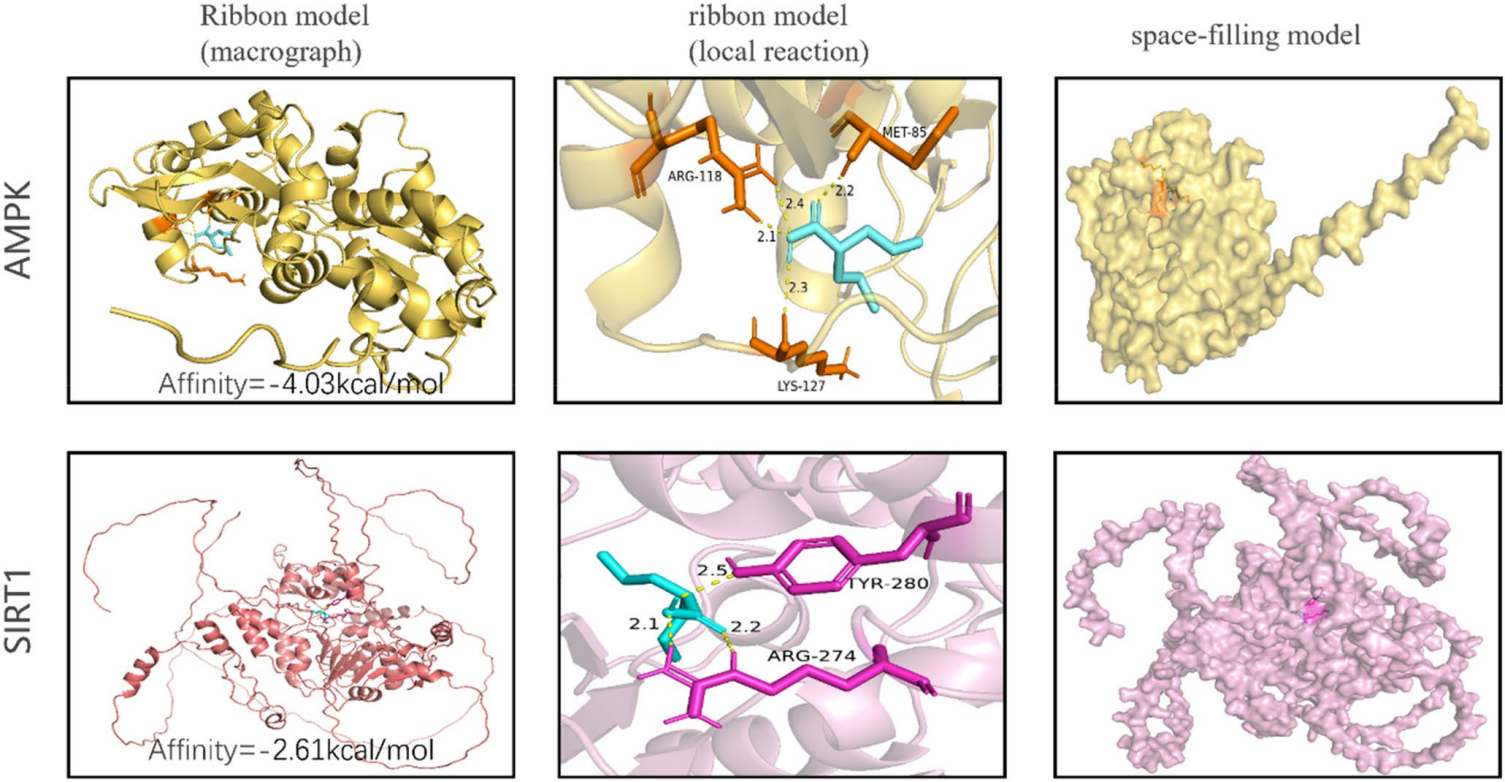
Molecular docking analysis demonstrates strong binding interactions between VPA and the AMPK/SIRT1 pathway. (A) Ribbon diagrams illustrating the overall binding conformation and local interaction details between VPA and (i) AMPK or (ii) SIRT1. Key interacting residues are highlighted. (B) Space‐filling models showing the three‐dimensional binding configuration of VPA within the active sites of (i) AMPK and (ii) SIRT1. Quantitative analysis of binding affinities, demonstrating favorable hydrogen bond formation energies of −4.03 kcal/mol for VPA‐AMPK and −2.61 kcal/mol for VPA‐SIRT1 complexes. These results indicate that VPA can effectively bind to the active sites of both AMPK and SIRT1, suggesting its potential role in modulating the AMPK/SIRT1 signaling pathway.

To investigate the molecular interactions between VPA and key components of the AMPK/SIRT1 pathway, we performed computational molecular docking simulations. Our analysis demonstrated successful docking of VPA to the binding pockets of both AMPK and SIRT1 proteins. The binding configurations were visualized using ribbon diagrams to illustrate overall structural interactions and space‐filling models to detail 3D binding conformations. Quantitative analysis revealed favorable binding energies, with hydrogen bond formation energies of −4.03 kcal/mol for VPA‐AMPK and −2.61 kcal/mol for VPA–SIRT1 complexes (Figure 5). These computational findings strongly suggest that VPA exhibits significant binding affinity for both AMPK and SIRT1, supporting its potential role in modulating the AMPK/SIRT1 signaling pathway.

### 3.6 VPA Protects BMSCs From Ferroptosis via the AMPK/SIRT1 Pathway

**FIGURE 6:**
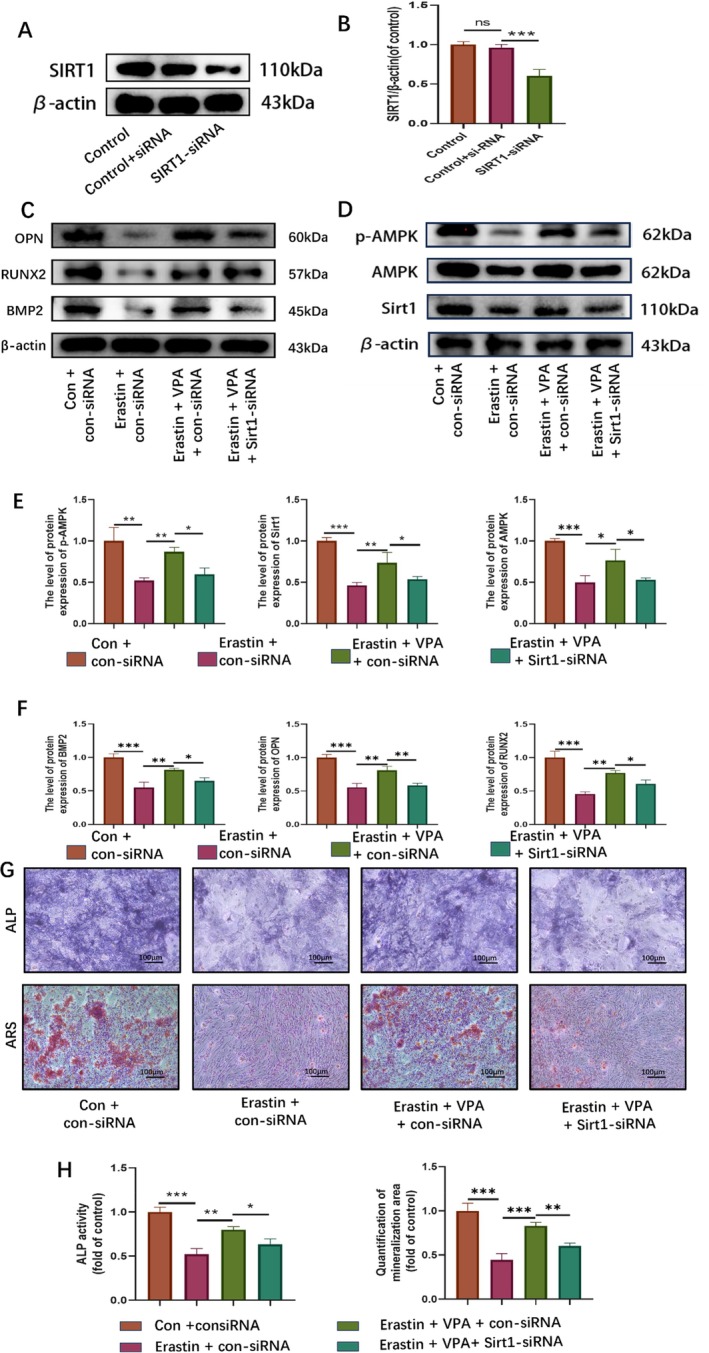
Inhibition of Ferroptosis and Enhancement of Osteogenic Capacity by VPA Are Regulated through Sirt1‐siRNA. (A, B) Western blot analysis of SIRT1 protein expression levels in BMSCs across different treatment groups. (C, F) Western blot detection of osteogenic markers (BMP2, RUNX2, and OPN) protein expression in BMSCs under various treatments. (D, E) Western blot evaluation of p‐AMPK, AMPK, and SIRT1 protein expression in treated BMSCs. (G) Representative images of alkaline phosphatase (ALP) staining at Day 7 and Alizarin Red S (ARS) staining at Day 21 following osteogenic induction in different groups. (H) Quantitative analysis of ALP activity and mineralization area using microplate reader. Data are presented as mean ± SD (*n* = 3 independent experiments). *p < 0.05, **p < 0.01, ***p < 0.001 versus Con + con‐siRNA group.

To explore the role of SIRT1 in Erastin‐induced ferroptosis, SIRT1 expression was silenced using SIRT1‐siRNA. Western blot analysis showed a marked reduction in SIRT1 expression after transfection (Figure 6A,B). Under SIRT1 knockdown conditions, the expression levels of key osteogenic markers (OPN, RUNX2, and BMP2) were significantly diminished, indicating a pronounced decline in osteogenesis (Figure 6C,E). Additionally, SIRT1‐siRNA inhibited the activation of the AMPK/SIRT1 pathway by VPA (Figure 6D,F). Alkaline phosphatase (ALP) activity and Alizarin Red staining further confirmed that SIRT1 knockdown counteracted the stimulatory effects of VPA on BMSC osteogenic differentiation (Figure 6G,H).

### 3.7 Establishment and Validation of the OVX Rat Model

**FIGURE 7:**
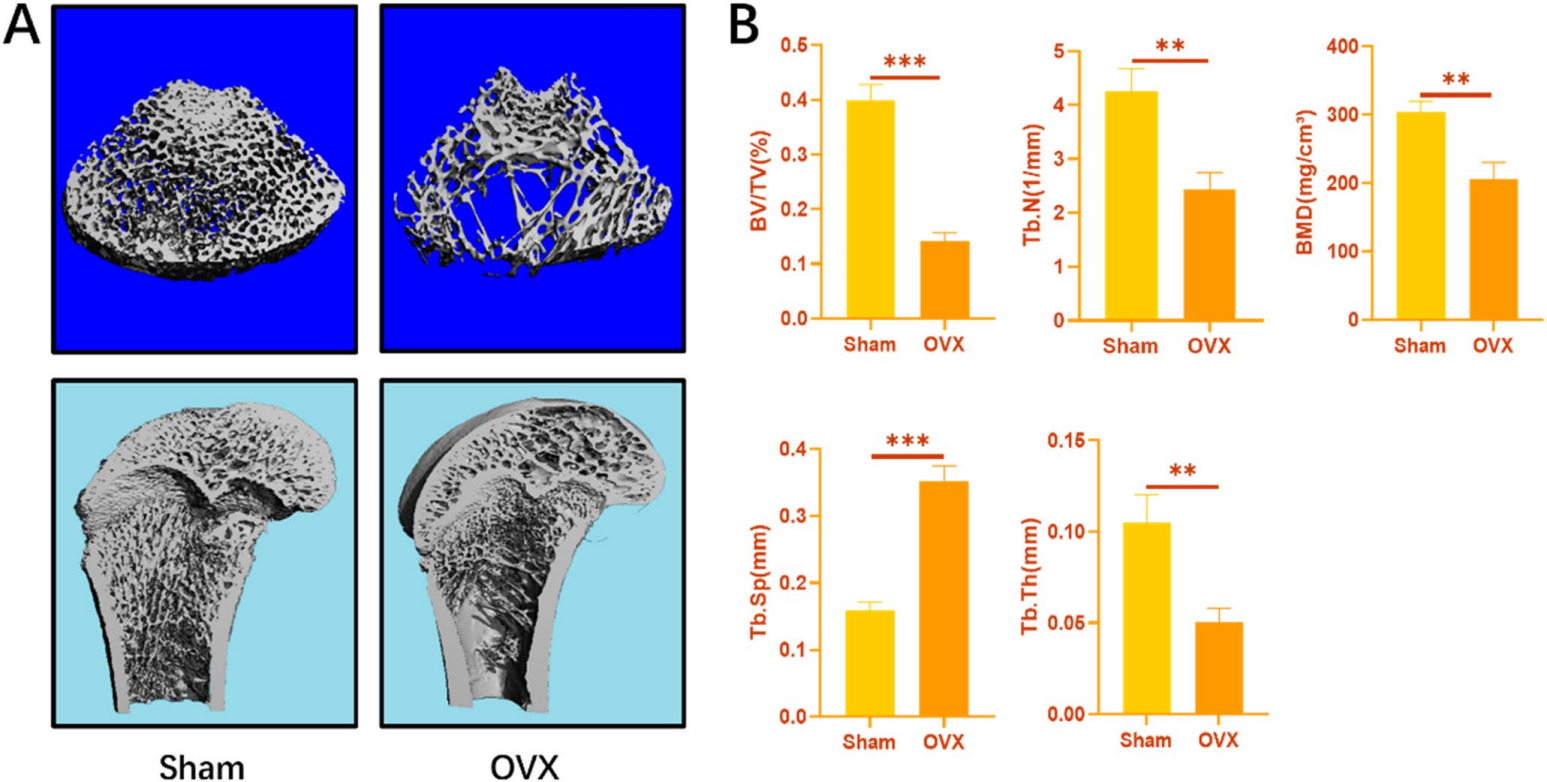
Establishment of OVX rat model. (A) Representative three‐dimensional reconstructed micro‐computed tomography (micro‐CT) images of distal femurs from sham‐operated (sham) and ovariectomized (OVX) groups. (B) Quantitative micro‐CT analysis of trabecular microarchitecture parameters in distal femurs: BV/TV: Bone volume fraction; Tb.N: Trabecular number; BMD: Bone mineral density; Tb.Sp: Trabecular separation; Tb.Th: Trabecular thickness. Data are presented as mean ± SD from three independent experiments. **p < 0.01, ***p < 0.001 versus sham group.

Micro‐CT imaging and quantitative evaluation of femora from rats were conducted. The reconstructed images revealed a decrease in trabecular bone count and disruptions in trabecular continuity within the distal femur region of OVX rats, as depicted in Figure 7A. Quantitative analysis demonstrated that the OVX group exhibited significantly reduced bone volume fraction (BV/TV), trabecular number (Tb.N), BMD, and trabecular thickness (Tb.Th), in contrast to the Sham group. Conversely, trabecular separation (Tb.Sp) was increased (Figure 7B). These findings confirm the successful establishment of the osteoporotic rat model.

### 3.8 VPA Significantly Improves Bone Quality and Promotes Bone Integration in OVX Rats

**FIGURE 8:**
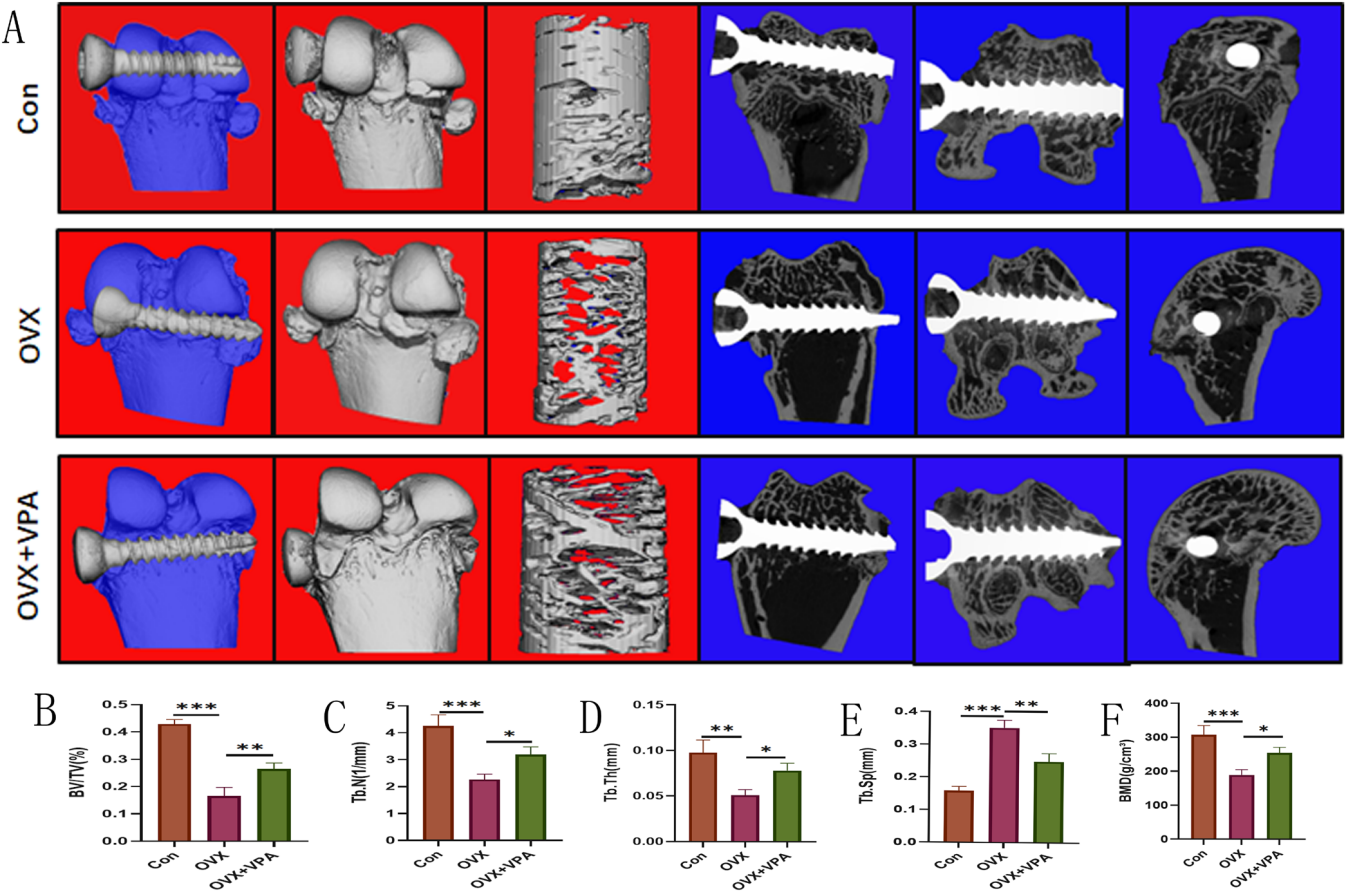
Effects of VPA on bone quality and osseointegration in OVX rats. (A) Representative three‐dimensional reconstructed micro‐CT images of titanium rods and surrounding tissues in distal femurs. (B–F) Quantitative analysis of trabecular microarchitecture parameters in the region of interest (ROI) surrounding the implants. Data represent mean ± SD from three independent biological replicates. *p < 0.05, **p < 0.01, ***p < 0.001 versus OVX group.

The impact of VPA on the quality of trabecular bone and bone integration was assessed in a rat model of osteoporosis. Figure 7 showcases the 3D reconstructions of the femora using micro‐CT technology, revealing detailed insights into the trabecular bone structure and biomechanical properties. The results demonstrated that, following an 8‐week period of VPA administration, the trabecular structure in the distal femur of OVX rats exhibited enhancement, with an expansion observed in the contact area between the titanium screw and adjacent bone tissue (Figure 8A). More precisely, VPA treatment led to a decrease in BV/TV, Tb.N, Tb.Th, and BMD, whereas Tb.Sp exhibited an increase (Figure 8B–F).

**FIGURE 9:**
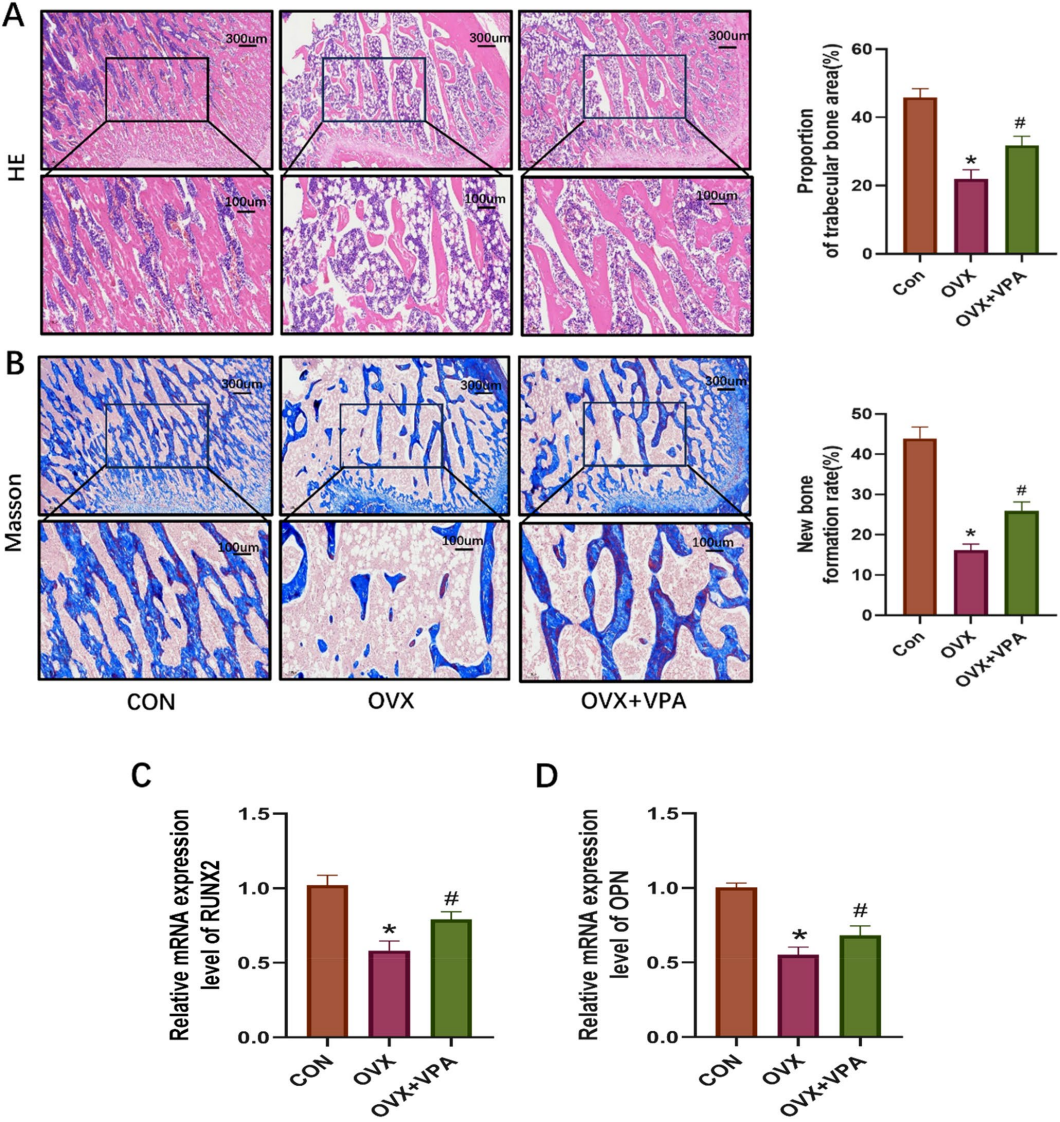
Protective effect of VPA on trabecular structure and enhancement of osteogenic gene expression (RUNX2 and OPN). (A) Representative images and quantitative analysis of hematoxylin and eosin (H&E) staining in distal femoral sections. (B) Representative images and quantitative assessment of Masson's trichrome staining in distal femoral sections. (C) Relative mRNA expression levels of RUNX2 in bone tissue. (D) Relative mRNA expression levels of osteopontin (OPN) in bone tissue. Data are presented as mean ± SD from three independent biological replicates. *p < 0.05 versus control group; ^#^ *p* < 0.05 versus OVX group.

To further examine the effects of VPA, H&E and Masson staining were performed. The results indicated that, compared to the OVX group, the OVX + VPA group showed denser trabeculae with improved structural integrity (Figure 9A) and a greater presence of newly formed bone tissue and collagen fibers (Figure 9B).

RT‐qPCR analysis revealed that RUNX2 and OPN mRNA expression levels were higher in the OVX + VPA group compared to the OVX group, although they remained lower than those observed in the Con group (Figure 9C,D). We hypothesize that this may be linked to an accelerated bone formation process, which could contribute to the enhancement of bone density and restoration of bone structure.

### 3.9 VPA Inhibits Ferroptosis in OVX Rats

**FIGURE 10:**
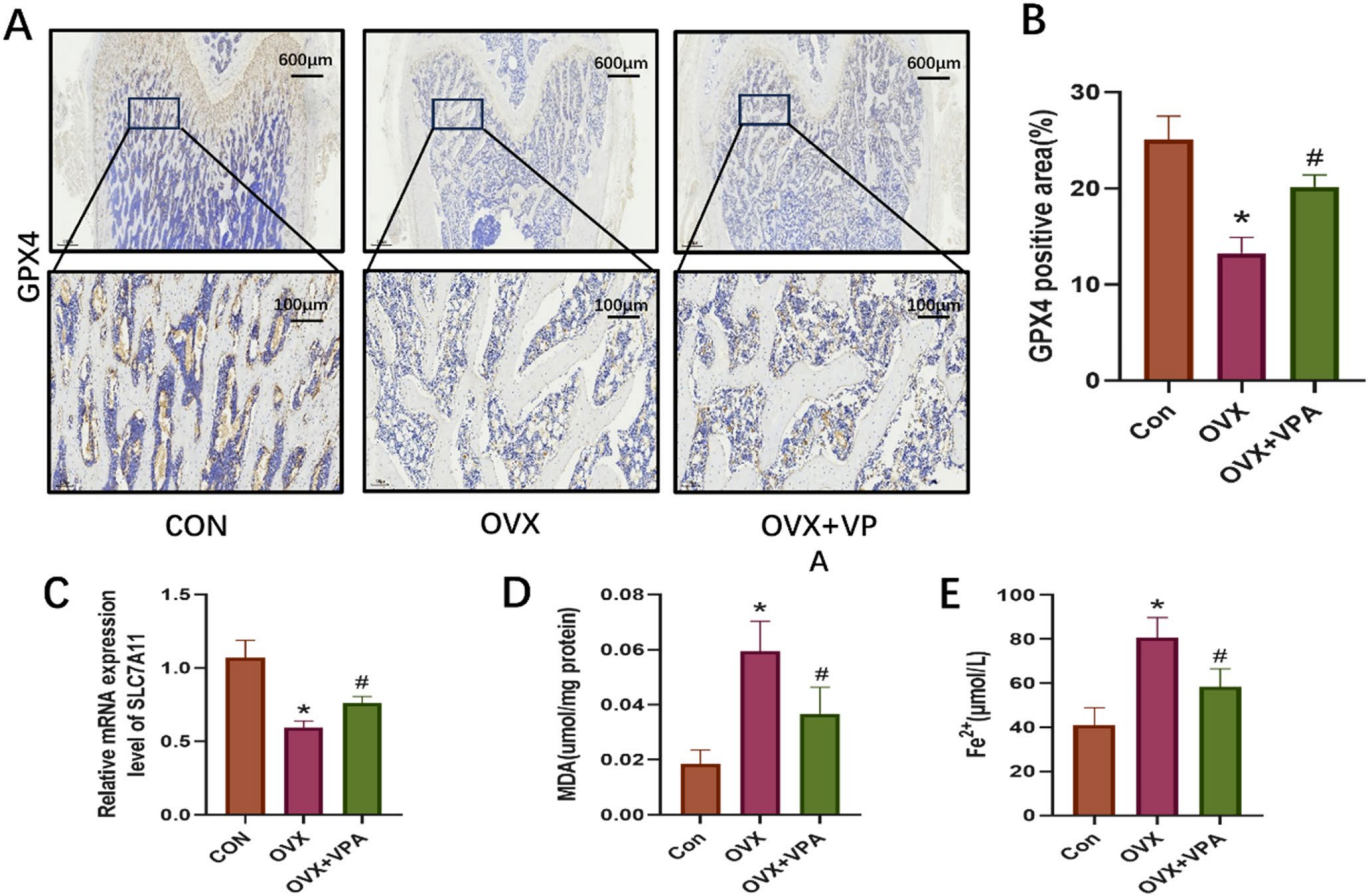
Impact of VPA on GPX4 and SLC7A11 expression levels and the reduction of MDA and Fe^2+^ content (A) VPA partially mitigated the OVX‐induced reduction in GPX4 protein expression. (B) Quantitative analysis of GPX4 protein expression. (C) VPA increased the OVX‐induced decrease in SLC7A11 mRNA expression. (D) VPA reduced the MDA content in bone tissue. (E) VPA reduced the Fe2+ content in bone tissue. *p < 0.05 compared to the Con group, ^#^ *p* < 0.05 compared to the OVX group. Data are presented as mean ± SD from three independent biological replicates.

Excessive iron promotes the production of ROS and exacerbates lipid peroxidation [8]. On the other hand, activation of GPX4 and SLC7A11 can mitigate iron‐dependent damage by inhibiting lipid peroxidation [22]. As illustrated in Figure 10, ferroptosis was evident in OVX rats. Compared to the Con group, the OVX group exhibited significantly reduced GPX4 and SLC7A11 expression levels, while MDA and Fe^2+^ levels were substantially increased. However, VPA treatment in OVX rats markedly increased GPX4 and SLC7A11 expression levels in bone tissue (Figure 10A–C) while simultaneously lowering Fe^2+^ and MDA levels (Figure 10D,E).These findings indicate that ferroptosis occurs in OVX rats and that VPA effectively inhibits its progression.

### 3.10 VPA Activates the AMPK/Sirt1 Signaling Pathway in OVX Rats

**FIGURE 11:**
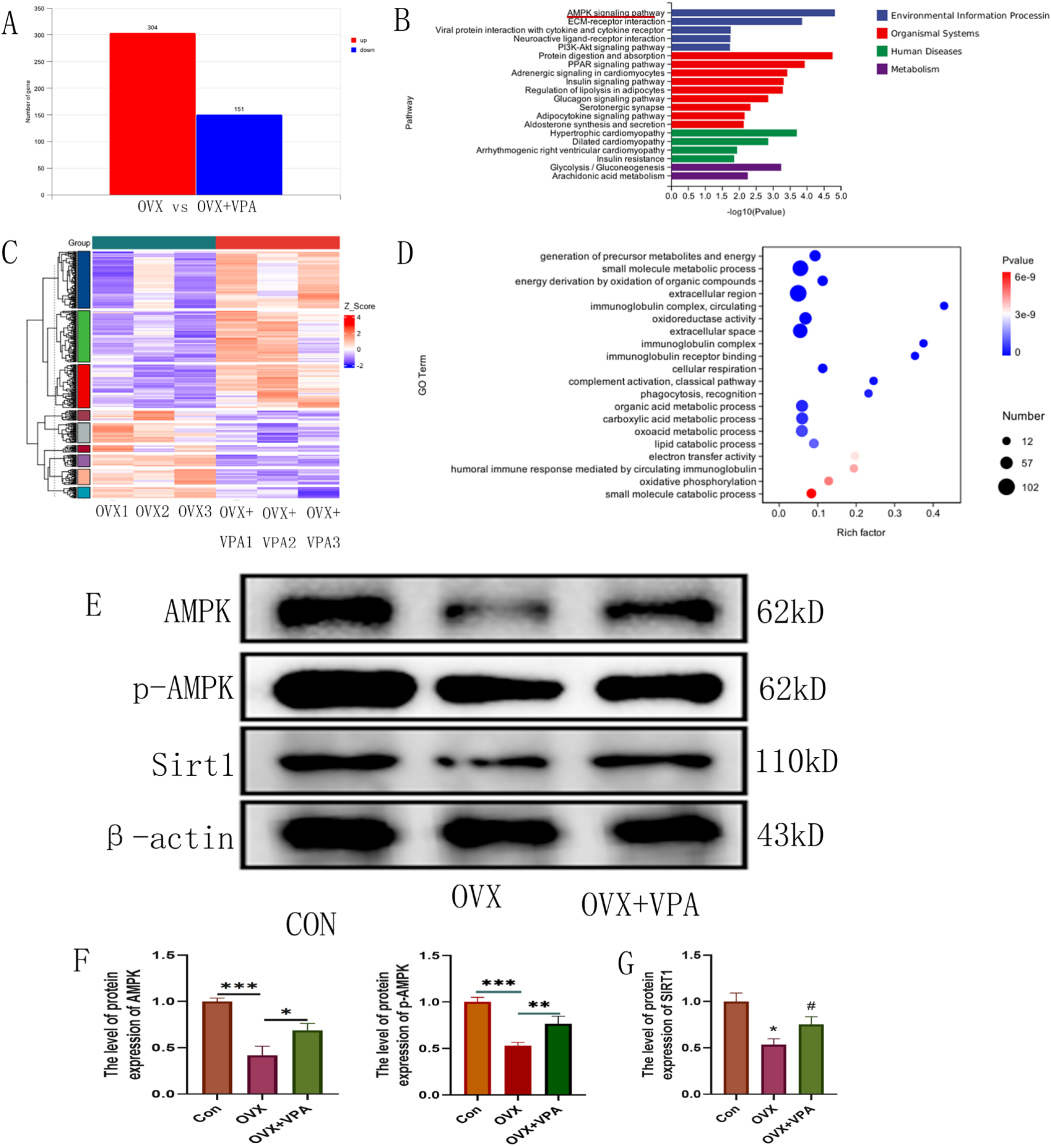
VPA activates the AMPK/SIRT1 signaling pathway. (A) Upregulated and downregulated differentially expressed genes (DEGs) were identified through RNA sequencing analysis. (B) KEGG pathway enrichment analysis revealed significant changes in cellular pathways. (C) Cluster heatmap analysis showed significant gene alterations in the OVX + VPA group compared to the OVX group. (D) GO enrichment analysis revealed the main biological functions of the differentially expressed genes. (E) VPA increased the expression levels of AMPK, p‐AMPK, and Sirt1 proteins in OVX rats. (F) Quantitative analysis of AMPK and p‐AMOK protein expression levels. (G) Quantitative analysis of Sirt1 protein expression levels. *p < 0.05 compared to the Con group, ^#^ *p* < 0.05 compared to the OVX group. Data are presented as mean ± SD from three independent biological replicates.

We utilized RNA sequencing to analyze the expression profiles of femurs in the OVX and OVX + VPA groups. Differential expression analysis identified a total of 304 upregulated genes and 151 downregulated genes (Figure 11A). Heatmap results showed significant changes in gene expression in the VPA treatment group (Figure 11C). GO enrichment analysis (Figure 11D) revealed the main biological functions of the differentially expressed genes. Furthermore, KEGG pathway enrichment analysis (Figure 11B) was conducted to identify significantly enriched pathways among differentially expressed genes. Analysis identified significant enrichment in AMPK‐related pathways in this study, suggesting that VPA may prevent ferroptosis by alleviating oxidative stress through activation of the AMPK pathway. Sirt1, as a downstream effector of GPX4 and SLC7A11, exerts protective effects by modulating lipid metabolism and ferroptosis, as evidenced by studies showing that SLC7A11 stabilizes the system XC and prevents iron‐dependent cell death in acute liver injury. AMPK stimulates Sirt1 by elevating NAD^+^ levels, while Sirt1 regulates AMPK through deacetylation. Compared with the Con group, the expression levels of AMPK, p‐AMPK, and Sirt1 were significantly decreased in the OVX group; however, VPA treatment significantly restored this downregulation (Figure 11E–G).

## 4 Discussion

In this study, we established a PMOP model in female rats through bilateral ovariectomy (OVX) and induced ferroptosis in BMSCs in vitro using Erastin (20 μM). The results demonstrated that VPA improved bone quality in OVX rats, promoted bone integration, enhanced bone microarchitecture, and upregulated the expression of osteogenic genes RUNX2 and OPN. We discovered, for the first time, that VPA elevated the expression levels of GPX4 and SLC7A11, decreased MDA and Fe^2+^ concentrations, and initiated the AMPK/SIRT1 signaling pathway. Furthermore, in vitro experiments validated that VPA facilitated osteoblast differentiation and mineralized nodule formation through the activation of the AMPK/SIRT1 pathway. Moreover, these effects were reversed following knockdown of SIRT1 expression using SIRT1‐siRNA. Our study provides the first evidence that VPA protects OVX rats from ferroptosis and enhances bone integration by activating the AMPK/SIRT1 signaling pathway.

The impact of ferroptosis on PMOP remains a relatively underexplored area. This unique form of cell death arises from disruptions in iron metabolism, imbalances in antioxidant defense mechanisms, and the accumulation of lipid peroxidation products [23]. These are regulated by key factors such as SLC7A11 and GPX4 [24]. Therefore, the levels of SLC7A11 and GPX4 within cells may serve as potential indicators for ferroptosis. There is substantial evidence indicating that ferroptosis plays a significant role in a variety of pathological conditions, including cancer, ischemic organ damage, and neurodegenerative diseases [25, 26]. This evidence suggests that ferroptosis could be harnessed as a therapeutic strategy, either as a standalone treatment or in combination with other targeted therapies. Under TEM, Erastin treatment induced significant alterations in mitochondrial morphology in BMSCs, characterized by increased mitochondrial volume, elevated membrane density, and a reduction in cristae number. Our study found that the ferroptosis inducer Erastin downregulates SLC7A11 and GPX4 in BMSCs, accelerating mitochondrial dysfunction. Consistent with this, the expression of GPX4 in the femur of OVX rats was significantly lower than that in the control group, suggesting that ferroptosis in BMSCs may be a contributing factor to the impaired bone integration in OVX rats.

To elucidate the molecular mechanisms through which VPA enhances bone integration in OVX rats, we performed RNA sequencing analysis in conjunction with expression profile assessment. In the differential expression results, we identified 304 upregulated genes and 151 downregulated differentially expressed genes (DEGs). We then performed GO enrichment analysis and KEGG pathway analysis on the DEGs to determine the main biological functions of the differentially expressed genes and discovered that the AMPK signaling pathway is closely associated with the protective effects of VPA. The interaction between AMPK and SIRT1 plays a crucial role in regulating energy metabolism, antioxidant response, and ferroptosis [27]. AMPK and SIRT1 are closely associated with PMOP, antioxidant effects, and ferroptosis. Studies have indicated that resveratrol alleviates estrogen deficiency‐induced cardiac dysfunction by activating the AMPK/SIRT1/Nrf2 signaling pathway [28]. Yu et al. pointed out that Schisandrin B inhibits LPS‐induced endometritis and alleviates ferroptosis by modulating the AMPK signaling pathway [29]. These studies suggest that the AMPK/SIRT1 pathway plays a crucial role in estrogen deficiency‐related ferroptosis.

BMP2 is widely regarded as a key factor in bone formation, as it can induce the differentiation of stem cells into osteoblasts and promote the deposition of bone matrix [30]. As an important member of the TGF‐β family, BMP2 binds to receptors on the cell membrane, activating the Smad signaling pathway, which in turn promotes the expression and activity of Runx2 [31]. Runx2 is a core transcription factor in the osteoblast differentiation process. It is not only regulated by the BMP signaling pathway but also reciprocally regulates the expression of BMPs [32]. OPN is a key marker in the osteoblast differentiation process. Runx2 promotes the transcription of OPN by binding to the promoter region of the OPN gene [33]. This study first demonstrates that VPA significantly increases the expression of BMP2, Runx2, and OPN, thereby restoring the osteogenic capacity of Erastin‐treated BMSCs. Furthermore, the osteogenic effect of VPA was inhibited by Sirt1‐siRNA, indicating that the AMPK/SIRT1 pathway is an important mediator of the osteogenic effects of VPA.

Recent studies have revealed that long‐term VPA use exerts dual effects on skeletal health. On one hand, VPA is strongly associated with reduced BMD and an increased risk of osteoporotic fractures. On the other hand, VPA exhibits osteogenic potential under specific conditions. Notably, the bone‐protective effects of VPA appear to be dose‐ and time‐dependent. This review focuses on the osteogenic efficacy of VPA. VPA, an inhibitor of histone deacetylase, has shown notable therapeutic benefits in anti‐inflammatory, anti‐apoptotic, antioxidative stress, and neuroprotective pathways [34, 35]. VPA can inhibit endoplasmic reticulum stress and reduce ferroptosis following traumatic brain injury [36]. Li et al. found that VPA eliminates ferroptosis in epileptic seizures by inhibiting lysyl oxidase [37]. Our previous studies have shown that VPA exerts a protective effect against bone loss in OVX rats [38], but its underlying mechanisms remain to be further explored. The findings of this study reveal that VPA significantly reverses Erastin‐induced ferroptosis in BMSCs, mitigates mitochondrial oxidative stress damage, and upregulates the expression of SLC7A11 and GPX4, as well as promotes osteogenic differentiation and mineralization. Furthermore, VPA augmented the expression levels of key osteogenesis‐related factors, namely OPN, Runx2, and BMP2. Moreover, using Sirt1‐siRNA to inhibit SIRT1 function significantly reduced the osteogenic‐enhancing effects of VPA, highlighting the dependency of VPA on the AMPK/SIRT1 signaling cascade. In vivo studies further confirmed that VPA enhanced the bone integration of titanium implants and increased the expression levels of SIRT1, AMPK, and osteogenic‐related factors (such as OPN, Runx2, and BMP2) in the distal femur. Furthermore, immunohistochemical analysis revealed that VPA markedly enhanced the expression levels of GPX4. The findings indicate that VPA has the potential to suppress ferroptosis in BMSCs via the AMPK/SIRT1 signaling cascade, leading to an augmentation in bone mass and facilitating bone incorporation at the titanium screw site in the distal femur (Figure 11).

**FIGURE 12:**
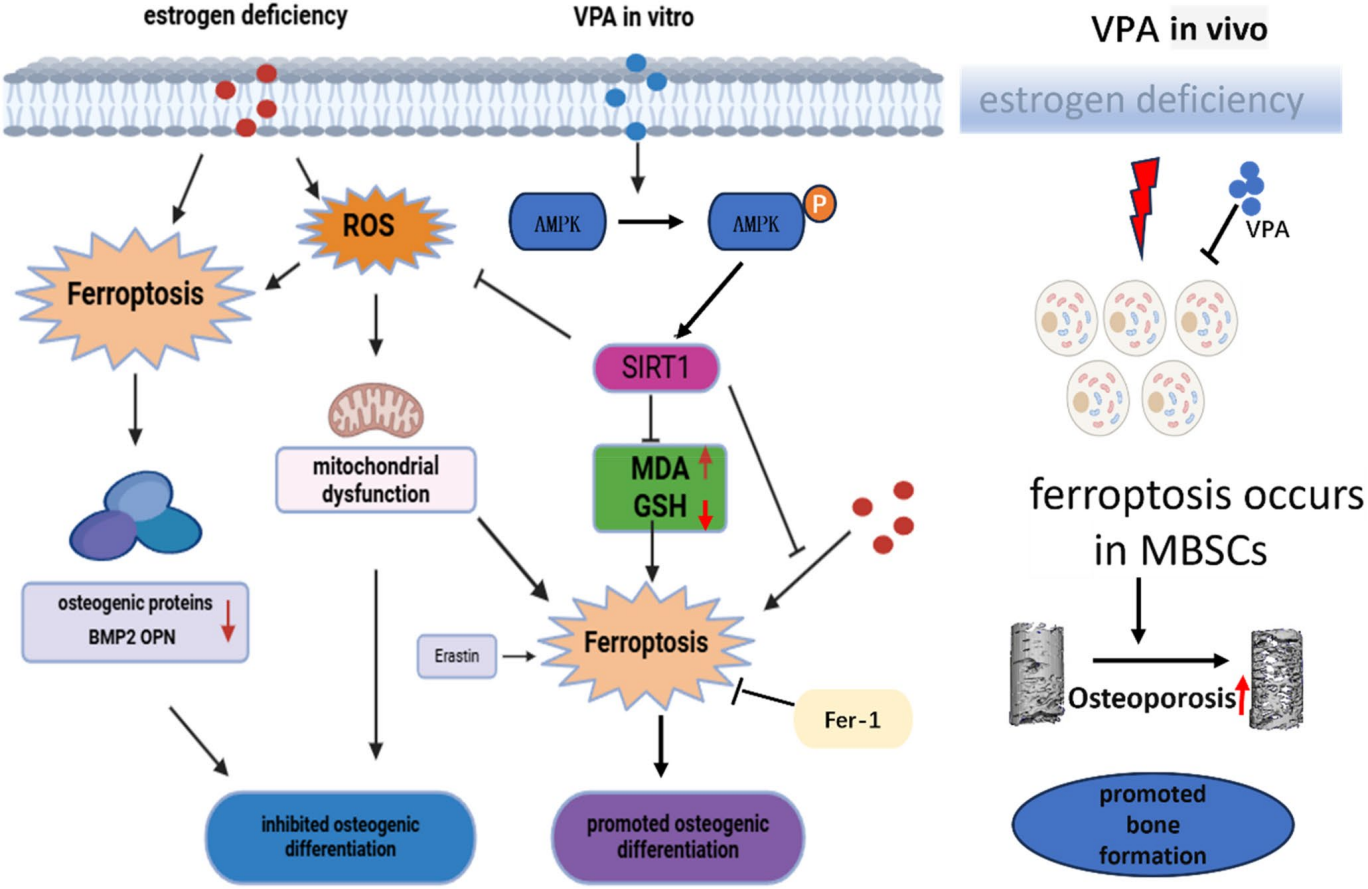
Mechanistic diagram of VPA's action on Erastin‐induced BMSCs.

However, this study has a limitation: only a single dose of VPA (150 mg/kg, administered every other day by gavage) was used, and no dose–response gradient was established. Consequently, the optimal effective window remains undefined. Future work should include multiple dose groups (50–500 mg/kg) and varied treatment durations to construct comprehensive dose–response curves for bone microarchitecture (micro‐CT), ferroptosis‐related biomarkers, and systemic toxicity, thereby identifying the safest and most effective dosage for clinical translation (Figure 12).

Our findings suggest that VPA may regulate ferroptosis in BMSCs by activating the AMPK/SIRT1 signaling pathway, thereby promoting bone integration in OVX rats. This discovery provides both a theoretical foundation and practical guidance for the design of prosthetics and drug development aimed at enhancing bone integration. It holds promise for achieving faster and more stable bone integration outcomes, ultimately improving the quality of life for patients.

Valproate (VPA) has a well‐documented safety record in long‐term anticonvulsant use. Our data indicate that pharmacological activation of the AMPK/SIRT1‐GPX4 axis suppresses osteoblastic ferroptosis and accelerates implant osseointegration in OVX rats, providing a rationale for repurposing VPA in PMOP. Three translational gaps should be addressed before human testing: (1) dose‐finding—escalating VPA exposure (50–500 mg kg^−1^ equivalents) in hip‐fracture patients scheduled for arthroplasty, while serially measuring intra‐osseous GPX4, systemic iron indices, and liver enzymes to define the minimal effective concentration; (2) local delivery—engineering VPA‐eluting titanium coatings or injectable microspheres to confine drug levels to the surgical bed and minimize neurohepatic adverse events; and (3) patient enrichment—selecting individuals with high marrow iron burden or low GPX4 content to magnify therapeutic signal. A single‐arm phase I safety study should precede a randomized controlled trial with “implant fixation” and “bone ferroptosis biomarkers” as co‐primary end‐points, laying the evidentiary groundwork for VPA as an adjuvant therapy to enhance prosthetic integration in osteoporotic subjects. And, all implants were placed 1–3 mm below the growth plate, where residual cartilage and high‐turnover trabeculae may overestimate bone formation. Future studies should compare sites distant from the growth plate (e.g., diaphyseal‐metaphyseal junction) to clarify VPA's effect on mature cortical bone integration.

## Funding

This research was supported by Anhui Province Higher Education Science Research Project (Grant No. 2023AH040265), Anhui Clinical Medical Research Transformation Project (Grant No. 202304295107020007), Talented Scholars of Wannan Medical College (Grant No. YR202226), Technology Mountaineering Program of Yijishan Hospital, and Wannan Medical College (Grant No. PF2019005), Research project of Anhui Provincial Health Commission (AHWJ2023A10149).

## Ethics Statement

All procedures were approved by Scientific Research and New Technology of Wannan Medical College Yijishan Hospital IRB (2021‐71). This study did not involve human participants and therefore did not require ethical approval.

## Conflicts of Interest

The authors declare no conflicts of interest.

## Data Availability

The data that support the findings of this study are available from the corresponding author upon reasonable request.
